# Statistical Analysis of Metal Chelating Activity of *Centella asiatica* and *Erythroxylum cuneatum* Using Response Surface Methodology

**DOI:** 10.1155/2013/137851

**Published:** 2013-02-27

**Authors:** R. J. Mohd Salim, M. I. Adenan, A. Amid, M. H. Jauri, A. S. Sued

**Affiliations:** ^1^Natural Product Division, Drug Discovery Centre (DDC), Forest Research Institute Malaysia (FRIM), Jalan Kepong, Selangor Darul Ehsan, 52109 Kepong, Malaysia; ^2^Malaysian Institute of Pharmaceuticals and Nutraceuticals, Ministry of Science, Technology and Innovation, USM, 10 Persiaran Bukit Jambul, 11900 Bukit Jambul, Malaysia; ^3^Department of Biotechnology Engineering, International Islamic University Malaysia, Gombak, P.O. Box 10, 50728 Kuala Lumpur, Malaysia

## Abstract

The purpose of the study is to evaluate the relationship between the extraction parameters and the metal chelating activity of *Centella asiatica* (CA) and *Erythroxylum cuneatum* (EC). The response surface methodology was used to optimize the extraction parameters of methanolic extract of CA and EC with respect to the metal chelating activity. For CA, Run 17 gave optimum chelating activity with IC_50_ = 0.93 mg/mL at an extraction temperature of 25°C, speed of agitation at 200 rpm, ratio of plant material to solvent at 1 g : 45 mL and extraction time at 1.5 hour. As for EC, Run 13 with 60°C, 200 rpm, 1 g : 35 mL and 1 hour had metal chelating activity at IC_50_ = 0.3817 mg/mL. Both optimized extracts were further partitioned using a solvent system to evaluate the fraction responsible for the chelating activity of the plants. The hexane fraction of CA showed potential activity with chelating activity at IC_50_ = 0.090 and the ethyl acetate fraction of EC had IC_50_ = 0.120 mg/mL. The study showed that the response surface methodology helped to reduce the extraction time, temperature and agitation and subsequently improve the chelating activity of the plants in comparison to the conventional method.

## 1. Introduction

The knowledge and practice of traditional medicine are universal amongst the respected ethnic groups in each country. In Malaysia the benefits of herbal medicine are being conveyed down from one generation to another. Latif et al. [1] state that there are four sources of traditional Malaysian medicine, namely, Malay village medicine (including Orang Asli medicine), Chinese medicine (introduced from China), Indian medicine (introduced from India), and other forms of traditional medicine (including those introduced by the Javanese, Sumatrans, Arabs, Persians, Europeans, etc.).

*Centella asiatica* (CA) also locally known as pegaga is a crawling plant usually growing wildly in a humid climate around the globe. Its wide medicinal benefits include wound healing, enhancing memory, treating mental weariness [2], anti-inflammatory property [3], anticancer activity [4], antilipid peroxidativity [5], and free radical scavenger [6].

*Erythroxylum cuneatum forma cuneatum* (Miq.) Kurz (EC) is a genus of tropical flowering plants in the family of Erythroxylaceae [7]. While CAs are being well studied for their various medicinal fortunes *Erythroxylum cuneatum* (EC) on the other hand has a very limited report on its medicinal value. In Terengganu, the leaves are pounded and applied on the forehead of women after miscarriage. In Bunguran, Indonesia leaves are reported to be used in Sajur (vegetable soup) [8]. It is used in Thai traditional medicine for antifever purposes as well as an anti-inflammatory agent [9]. 

Neurodegenerative disease (ND) results from the deterioration of neurons which functionalize the intellectual and cognition ability of a human body [10]. Zecca et al. [11] reported that iron may engage in a mechanism involving many neurodegenerative disorders. It was deduced that, as the brain ages, iron accumulates in regions that are affected by Alzheimer and Parkinson diseases, diseases categorized under ND. Thus, it is the interest of the research to study the ability of CA and EC to chelate the metal iron and further optimize the extraction process of the plants with respect to their chelating activity. 

The extraction of plant material for example bioactive compounds can be affected by more than one factor such as particle size, extraction solvent, temperature, and time [12]. Response surface methodology is a software tool used to study the interaction that may occur between variable factors [13]. This statistical experimental design is a powerful tool that enables the extraction process conducted effectively by verifying the effects of operational factors and their interactions [14]. The traditional empirical methods only study a single factor at a time and fail to acknowledge the interaction that they have between each other [15].

## 2. Materials and Methods

### 2.1. Materials

*Centella asiatica* (CA) was purchased from local market, Pasar Borong Selayang, Selangor, and Erythroxylum cuneatum (EC) was collected from FRIM's compound. Methanol was purchased from Fisher Scientific, ethanol from J. Kollin Corporation, Germany, and hexane, ethyl acetate, and n-butanol from Merck, USA. All chemicals and solvents used were of analytical grade. Iron (II) sulfate heptahydrate (FeSO_4_) was a product of Aldrich, USA, 4,4′-[3-(2-pyridinyl)-1,2,4-triazine-5,6-diyl]bis also known as ferrozine from Aldrich, USA.

### 2.2. Methods

#### 2.2.1. Response Surface Methodology (RSM)

RSM was used to optimize the conditions for extraction of CA and EC to give the optimum metal chelating activity. A face-centered cube design (FCD) in RSM consisting of 30 experimental runs including six replications at the center point was chosen to evaluate the combined effect of the independent variables. Three levels were adopted and coded to low, center, and high levels. The experiments were performed in random order to minimize the effects of unexplained variability in the observed responses due to systematic errors [15]. The independent variables were temperature (°C), speed of rotation (rpm), ratio of raw material to solvent (g : mL), and time of extraction (h), while the response is the metal chelating activity reported in 1/IC_50_. As the software was meant to display the response at maximum, the inverse IC_50_ (1/IC_50_) was reported in this study so that the IC_50_ will be displayed at its optimum activity. 

The total of 30 runs designed by Design Expert by combining the parameters for extraction was shown in Table 1. The figures for each parameter were deduced from preliminary experiment. Each run was performed in triplicate. 

#### 2.2.2. Extraction Process

A constant weight of 2 g plants was used for all the 30 runs while adjusting accordingly to the ratio of methanol solvent that was needed in each run as outlined by Design Expert software. The plants were extracted in incubator shaker according to the combination parameters as given by each run. The extracts were then separated from the filtrate, and the methanol solvent was removed using rotary evaporator at 40°C and at a reduced pressure. The extracts from each run were then subjected to the metal chelating activity.

#### 2.2.3. Metal Chelating Activity

The assay was initiated by adding 250 *μ*L of 2.5 mM FeSO_4_ to 500 *μ*L sample solutions; CA and EC crude extracts were prepared in a series of concentrations. This mixture was vortexed briefly for 10 seconds before adding 250 *μ*L of 6 mM ferrozine. The mixture was vortexed again briefly for 10 seconds and allowed to equilibrate for 10 min at room temperature. The absorbance of the mixture (formation of the ferrous iron-ferrozine complex) was measured at 562 nm [16]. Sample solutions with appropriate dilutions were used as blanks. The ability of extracts to chelate ferrous ion was calculated relative to the control (consisting of iron and ferrozine only) using the following formula [17]:

(1) Chelating  effect  %=(Absorbance  of  control−Absorbance  of  sample)Absorbance  of  control ×100.

#### 2.2.4. Partitioning Process

The crude methanolic extracts were weighed to be 50 g and were suspended in water and then subjected to liquid-liquid partition by adding hexane, ethyl acetate, and n-butanol successively. The residual part that was suspended in water which was the water residue fraction [18] and the hexane, ethyl acetate, and n-butanol fraction were collected and subjected to metal chelating assay as described above.

## 3. Results and Discussion

### 3.1. Optimization of Extraction with respect to Metal Chelating Activity

The optimum 1/IC_50_ value for CA (referred to in Table 2) was 10.753 mg/mL (IC_50_ = 0.093 mg/mL) obtained in the combined interaction of the independent parameter at Run 17 with 25°C, 200 rpm, 1 g : 45 mL ratio, and for duration of 1.5 hour. 

Table 3 summarized the experimental results for EC. The optimum 1/IC_50_ value of 2.6196 mg/mL (IC_50_ = 0.3817 mg/mL) was obtained in Run 13 with temperature of 60°C, agitation at 200 rpm, and ratio of raw material to solvent 1 g : 35 mL ratio for extraction duration of 1 hour. 

### 3.2. Multiple Regression Analysis

The statistical model was developed by applying multiple regression analysis methods on using the experimental data for the metal chelating activity which is given in (2) for CA and in (3) for EC. The response function (*y*) measured the 1/IC_50_ value of the metal chelating activity of the crude extracts CA and EC. This value was related to the variables (*A*, *B*, *C*, *D*) by a second-degree polynomial using (2) and (3) which is displayed in terms of coded factors. The coefficients of the polynomial were represented by a constant term, *A*, *B*, *C*, and *D* (linear effects), *A* ^2^, *B* ^2^, *C* ^2^, and *D* ^2^ (quadratic effects), and *AB*, *AC*, *AD*, *BC*, *BD*, and *CD* (interaction effects). The analysis of variance (ANOVA) tables were generated, and the effect and regression coefficients of individual linear, quadratic, and interaction terms were determined. The significances of all terms in the polynomial were judged statistically by computing the *F*-value at a probability (*P*) of 0.001, 0.01, or 0.05. In this case *A*, *B*, *A* ^2^, *B* ^2^, *C* ^2^, *AB*, *AC*, *AD*, *BC*, *BD*, and *CD* are significant model terms. On the other hand, values greater than 0.1000 indicate that the model terms are not significant. The regression coefficients were then used to make statistical calculation to generate contour maps from the regression models:

(2) IC50=+1.79−1.16∗X1+0.58∗X2+0.051∗X3 +0.14∗X4+4.20∗X12−1.32∗X22 −1.18∗X320.31∗X42−0.77∗X1∗X2 −0.61∗X1∗X3−0.57∗X1∗X4 +0.43∗X2∗X3+1.10∗X2∗X4 +1.15∗X3∗X4,

(3) IC50=+2.29−0.15∗X1−0.023∗X2+0.022∗X3 −0.012∗X4−0.25∗X22+0.063∗X22 +0.022∗X32−0.48∗X42+0.10∗X1∗X2 −0.014∗X1∗X3−0.20∗X1∗X4 +0.026∗X2∗X3+3.750exp⁡−003∗X2∗X4 −0.010∗X3∗X4.

### 3.3. Fit Statistics for the Response

Some characteristics of the constructed model can be explained by details in Table 4 and Table 5. The statistical analysis indicates that the proposed model was adequate, possessing no significant lack of fit and with satisfactory values of the *R*-squared. The quality of fit of the polynomial model equation was expressed by the coefficient of determination (*R* ^2^, adjusted *R* ^2^, and adequate precision). *R* ^2^ is a measure of the amount of variation around the mean explained by the model and equal to 0.9569 (CA) and 0.9028 (EC). The closer the value of *R*-squared is to the unity, the better the empirical model fits the actual data. The smaller the value of *R*-squared is, the less relevant the dependent variables in the model have to explain the behavior variation [18] and [19]. The adjusted-*R* ^2^ is adjusted for the number of terms in the model. It decreases as the number of terms in the model increases, if those additional terms do not add value to the model. Adequate precision is a signal-to-noise ratio. It compares the range of the predicted values at the design points to the average prediction error. Ratios greater than four indicate adequate model discrimination. As for CA it was 21.064 whereas for EC it was 9.404. The standard deviation of 0.66 (CA) and 0.26 (EC) indicates that the model designed was acceptable with a minimum deviation. Coefficient of variation (C.V.) is the standard deviation expressed as a percentage of the mean which is 25.34% (CA) and 16.12 (EC). CV describes the extent to which the data were dispersed. The small values of CV give better reproducibility. In general, a high CV indicates that variation in the mean value is high and does not satisfactorily develop an adequate response model [20]. 

The predicted residual error sum of squares (PRESS) is a measure of model fitness to each point in the design which gave an amount of 46.29 (CA) and 16.12 (EC). 

## 4. Conclusion

The metal chelating activity of CA and EC was optimized using statistical analysis to improve the chelating activity of the both plants by varying the parameters for the extraction. It shows that the extraction parameters had been optimized (IC_50_ = 0.093 mg/mL at extraction temperature of 25°C, speed of agitation at 200 rpm, ratio of plant material to solvent at 1 g : 45 mL, and extraction time at 1.5 hour). As for EC, Run 13 with extraction temperature at 60°C, speed of agitation at 200 rpm, ratio of plant material to solvent at 1 g : 35 mL, and extraction time at 1 hour had metal chelating activity at IC_50_ = 0.3817 mg/mL. 

## Figures and Tables

**Table 1:** Parameters to be optimized using response surface methodology for CA and EC.

	CA	EC
Temperature (°C) (*X*_1_)	25, 30, 35	55, 60, 65
Speed (rpm) (*X*_2_)	100, 150, 200	150, 200, 250
Ratio (g : mL) (*X*_3_)	1 : 35, 1 : 40, 1 : 45	1 : 30, 1 : 35, 1 : 40
Time (min) (*X*_4_)	30, 60, 90	30, 60, 90

**Table 2:** Face centered, central composite design setting with the independent variables and their responses in CA.

Run number	*X*_1_	*X*_2_	*X*_3_	*X*_4_	*Y* 1/IC_50_	IC_50_
1	30	150	40	60	1.72	0.5814
2	30	150	40	60	1.429	0.6998
3	35	100	35	30	5.2	0.1923
4	30	150	40	60	1.55	0.6452
5	30	150	40	90	1.96	0.5102
6	35	200	35	30	2.4	0.4167
7	30	150	40	60	1.3426	0.7448
8	30	150	35	60	0.84	1.1905
9	30	150	40	30	1.57	0.6369
10	25	100	45	30	2.3	0.4348
11	35	200	45	90	2.703	0.3700
12	35	100	45	30	2.01	0.4975
13	25	100	35	30	4.6	0.2174
14	35	150	40	60	4.56	0.2193
15	30	200	40	60	1.399	0.7148
16	30	150	45	60	0.94	1.0638
17	25	200	45	90	10.753	0.0930
18	35	200	35	90	1.98	0.5051
19	30	150	40	60	1.49	0.6711
20	35	100	35	90	0.84	1.1905
21	30	150	40	60	1.49	0.6711
22	25	200	45	30	3	0.3333
23	35	200	45	30	0.29	3.4483
24	25	150	40	60	7.98	0.1253
25	25	100	45	90	3.49	0.2865
26	25	100	35	90	1.12	0.8929
27	25	200	35	30	4.167	0.2400
28	30	100	45	90	0.098	10.2041
29	35	100	45	90	0.93	1.0753
30	25	200	35	90	4.35	0.2299

**Table 3:** Face centered, central composite design setting with the independent variables and their responses in EC.

Run number	*X*_1_	*X*_2_	*X*_3_	*X*_4_	*Y* 1/IC_50_	IC_50_
1	60	200	35	60	2.4	0.4167
2	55	150	40	30	1.3	0.7692
3	55	250	30	90	1.4	0.7143
4	60	150	35	60	2.1	0.4762
5	60	200	30	60	1.5	0.6667
6	65	150	30	90	0.76	1.3158
7	65	150	30	30	1.18	0.8475
8	60	200	40	60	1.59	0.6289
9	60	200	35	60	2.6	0.3846
10	55	250	30	30	0.909	1.1001
11	65	150	40	30	1.1	0.9091
12	55	150	30	90	1.59	0.6289
13	60	200	35	60	2.62	0.3817
14	65	150	40	90	0.7	1.4286
15	60	200	35	60	2.57	0.3891
16	55	250	40	90	1.39	0.7194
17	60	200	35	90	1.57	0.6369
18	65	200	35	60	1.68	0.5952
19	60	200	35	60	2.56	0.3906
20	55	200	35	60	1.89	0.5291
21	60	200	35	30	1.55	0.6452
22	60	250	35	60	2.1	0.4762
23	65	250	30	90	0.833	1.2005
24	65	250	30	30	1.25	0.8000
25	60	200	35	60	2.56	0.3906
26	55	150	30	30	1.28	0.7813
27	65	250	40	30	1.36	0.7353
28	55	150	40	90	1.66	0.6024
29	65	250	40	90	0.906	1.1038
30	55	250	40	30	1.1	0.9091

**Table 4:** Fit statistics for the response of 1/IC_50_ value of CA.

Standard deviation	0.14	*R*-squared	0.9746
Mean	1.49	Adjusted *R*-squared	0.9509
C.V.	9.40	Predicted *R*-squared	0.8608
PRESS	1.62	Adequate precision	27.272

**Table 5:** Fit statistics for the response of 1/IC_50_ value of EC.

Standard deviation	0.26	*R*-squared	0.9028
Mean	1.60	Adjusted *R*-squared	0.8120
C.V.	16.12	Predicted *R*-squared	0.7243
PRESS	2.83	Adequate precision	9.404
